# Supplementary material for: Inguinal Fat Compensates Whole Body Metabolic Functionality in Partially Lipodystrophic Mice with Reduced PPARγ Expression
Source: Int J Mol Sci. 2023 Feb 15;24(4):3904. doi: 10.3390/ijms24043904 (PMC9966317; doi:10.3390/ijms24043904)
Supplement: Supplementary file 1 [file ijms-24-03904-s001.zip › ijms-2169895-supplementary.pdf]

Supplemental Figures

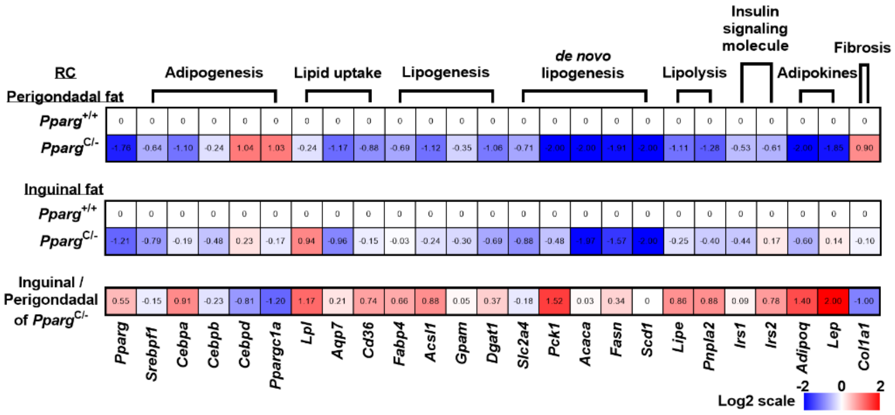

Supplemental Figure S1. mRNA levels of metabolic genes in adipose tissues of chow-fed *Pparg*$^{C/-}$ mice. The heat-map represents mRNA levels of metabolic genes analyzed by quantitative real-time PCR in perigonadal and inguinal fat of chow-fed *Pparg*$^{C/-}$ mice relative to *Pparg*$^{+/+}$ mice. The ratio of mRNA level of inguinal to perigonadal fat in *Pparg*$^{C/-}$ mice is also shown. The average log 2 fold-change values are inside squares. The log 2 expression scales represent the values in terms of logarithmic fold, blue (lowest), white (medium), and red (highest) expression levels. RC, regular chow.

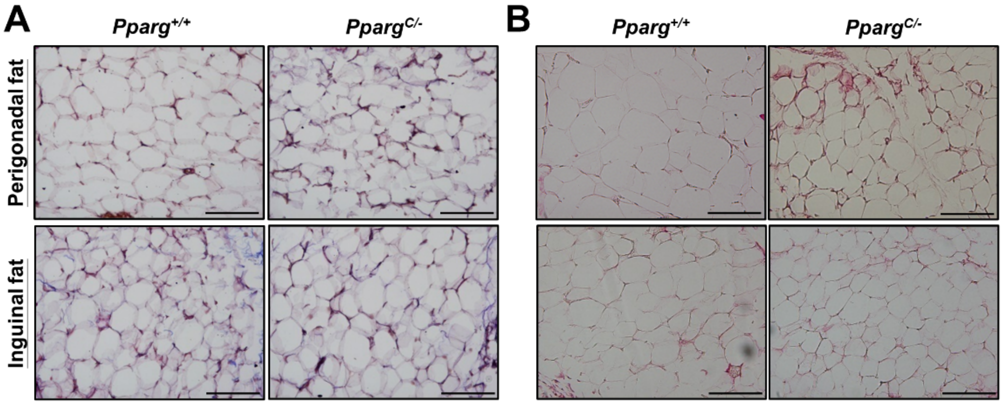

Supplemental Figure S2. Fibrosis staining of *Pparg*$^{C/-}$ **adipose tissue.** Masson's trichrome (A) and picrosirius red (B) staining in the perigonadal and inguinal fat of *Pparg*$^{C/-}$ mice and wild-type mice. Scale bar = 100 μm.

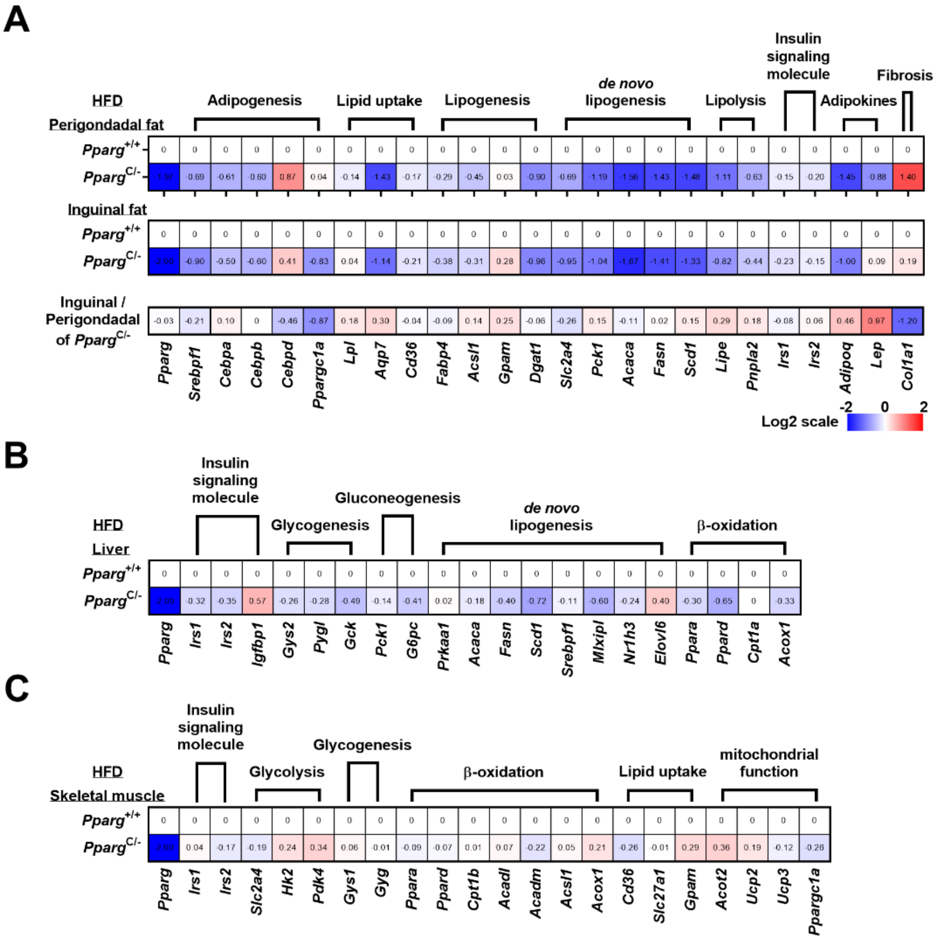

Supplemental Figure S3. mRNA levels of metabolic genes in metabolic tissues of HFD-fed *Pparg*$^{C/-}$ mice. The heat-map represents mRNA levels of metabolic genes analyzed by quantitative real-time PCR in perigonadal and inguinal fat (A), liver (B), and skeletal muscle (C) of HFD-fed *Pparg*$^{C/-}$ mice relative to *Pparg*$^{+/+}$ mice. The ratio of mRNA level of inguinal to perigonadal fat in *Pparg*$^{C/-}$ mice is also shown in (A). The average log 2 fold-change values are inside squares. The log 2 expression scales represent the values in terms of logarithmic fold, blue (lowest), white (medium), and red (highest) expression levels. HFD, high-fat diet.

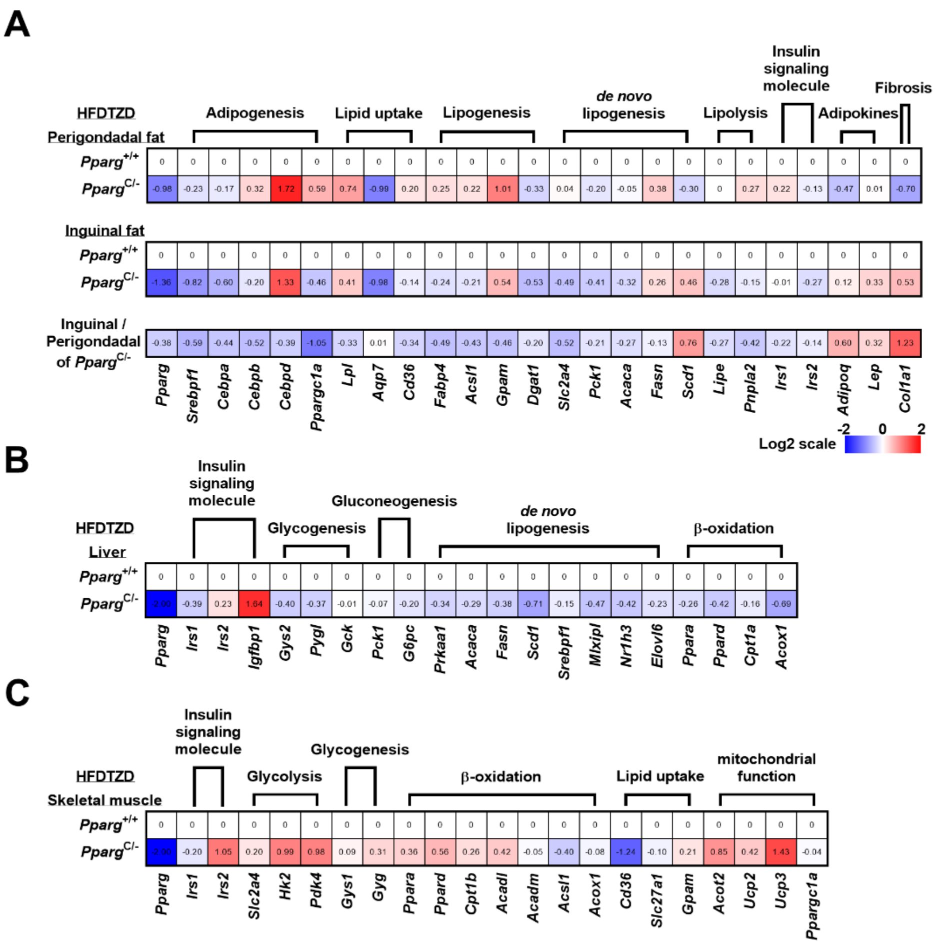

Supplemental Figure S4. mRNA levels of metabolic genes in metabolic tissues of HFDTZD-fed *Pparg*$^{C/-}$ mice. The heat-map represents mRNA levels of metabolic genes analyzed by quantitative real-time PCR in perigonadal and inguinal fat (A), liver (B), and skeletal muscle (C) of HFDTZD-fed *Pparg*$^{C/-}$ mice relative to *Pparg*$^{+/+}$ mice. The ratio of mRNA level of inguinal to perigonadal fat in *Pparg*$^{C/-}$ mice is also shown in (A). The average log 2 fold-change values are inside squares. The log 2 expression scales represent the values in terms of logarithmic fold, blue (lowest), white (medium), and red (highest) expression levels. HFDTZD, high-fat diet supplemented with TZD. This illustration emphasizes the genotype effect.

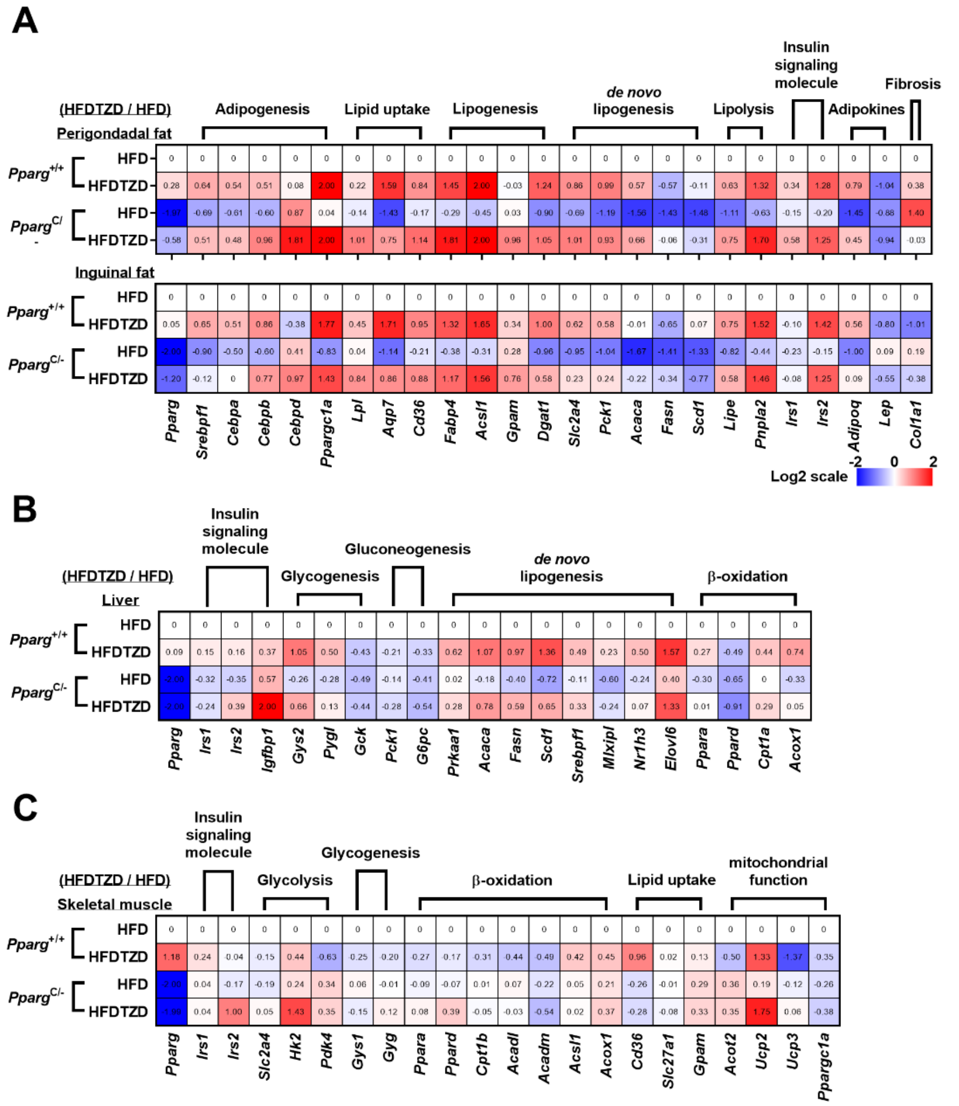

Supplemental Figure S5. mRNA levels of metabolic genes in metabolic tissues of both HFD-fed and HFDTZD-fed *Pparg*$^{C/-}$ mice. The heat-map represents mRNA levels of metabolic genes analyzed by quantitative real-time PCR in perigonadal and inguinal fat (A), liver (B), and skeletal muscle (C) of both HFD-fed and HFDTZD-fed *Pparg*$^{C/-}$ and *Pparg*$^{+/+}$ mice. The mRNA levels of HFD-fed *Pparg*$^{+/+}$ mice were used as the relative reference. The average log 2 fold-change values are inside squares. The log 2 expression scales represent the values in terms of logarithmic fold, blue (lowest), white (medium), and red (highest) expression levels. This illustration emphasizes the TZD effect.

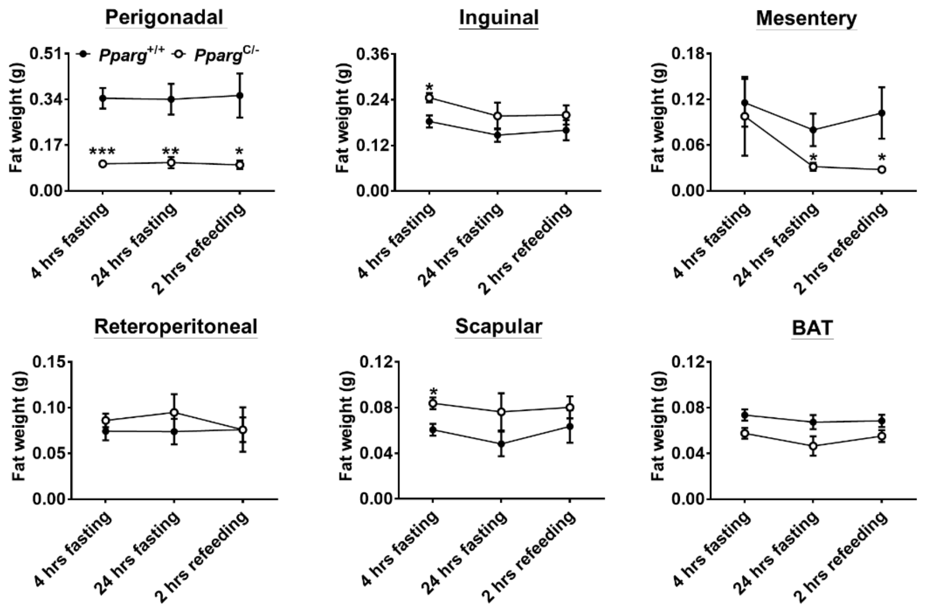

Supplemental Figure S6. Dynamics of fat weights during the 24 hrs of fasting and 2 hrs of refeeding course. The fat weight of various depots in *Pparg*$^{C/-}$ mice and *Pparg*$^{+/+}$ mice after 4 hrs fasting, 24 hrs fasting, or 2 hrs refeeding. * $p < 0.05$, ** $p < 0.01$, and *** $p < 0.001$ compared with *Pparg*$^{+/+}$ mice. This panel is associated with the experiment in Figure 2A

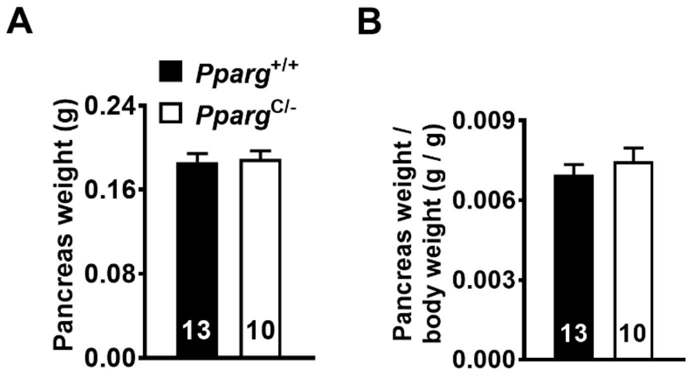

Supplemental Figure S7. Pancreas weight of *Pparg*$^{C/-}$ mice. Pancreas weight (A) and pancreas to body weight (B) in *Pparg*$^{+/+}$ and *Pparg*$^{C/-}$ mice.
